# Was the Nerve Regenerated?

**Published:** 1869-01

**Authors:** 


Was the Nerve Regenerated?—Two interesting cases have recently occurred at Strasbourg. In one, the details of which are strikingly similar to the case lately observed in Professor Richet's ward, we read that M. M. Bœckel and Hergott have had under their care a little boy, aged five years, who fell upon a shoemaker's knife, and received such a severe cut at the wrist that the radial artery, the median nerve, and the tendons of the various flexors were completely severed. A suture was applied to the flexor of the thumb and the superficial flexor of the fingers, but the median nerve was left untouched, as well as the other muscles; only the hand was bent upon the forearm, and maintained in that position. Complete and immediate cure was the very fortunate result; motility and sensibility were completely recovered. M. Chereau, whose able "Chronique Departementale" in the last number of *L'Union Medicale*, furnishes me with the above details, concludes his notice of the case with the following points of interrogation: Was the median nerve regenerated? or have the severed extremities remained apart? and in the latter case has the nervous fluid continued to circulate through the capricious meanderings of anastomoses?

---

The new French silver is apparently an improvement on the old-fashioned German silver, and is stated to be applicable to all the purposes to which ordinary commercial silver is applicable. It is composed of copper 56 per cent., nickel 40.64, tungsten 2.80, aluminum 0.56. It is a white, ductile, malleable, tenacious, sonorous alloy; its specific gravity is nine-tenths that of silver, its metallic lustre superior to that of silver, and its fusibility less, probably on account of the tungsten it contains.

